# Unusual association of non-anaplastic Wilms tumor and Cornelia de Lange syndrome: case report

**DOI:** 10.1186/s12885-016-2402-2

**Published:** 2016-06-13

**Authors:** Claudia Santoro, Andrea Apicella, Fiorina Casale, Angela La Manna, Martina Di Martino, Daniela Di Pinto, Cristiana Indolfi, Silverio Perrotta

**Affiliations:** Dipartimento della Donna, del Bambino e di Chirurgia Generale e Specialistica, Seconda Università degli Studi di Napoli, Via Luigi De Crecchio 4, Naples, 80138 Italy

**Keywords:** Cornelia de Lange Syndrome, Wilms tumor, *NIPLB*, Cohesins, Wnt pathway

## Abstract

**Background:**

Cornelia de Lange syndrome is the prototype for cohesinopathy disorders, which are characterized by defects in chromosome segregation. Kidney malformations, including nephrogenic rests, are common in Cornelia de Lange syndrome. Only one post-mortem case report has described an association between Wilms tumor and Cornelia de Lange syndrome. Here, we describe the first case of a living child with both diseases.

**Case presentation:**

Non-anaplastic triphasic nephroblastoma was diagnosed in a patient carrying a not yet reported mutation in *NIPBL* (c.4920 G > A). The patient had the typical facial appearance and intellectual disability associated with Cornelia de Lange syndrome in absence of limb involvement. The child’s kidneys were examined by ultrasound at 2 years of age to exclude kidney abnormalities associated with the syndrome. She underwent pre-operative chemotherapy and nephrectomy. Seven months later she was healthy and without residual detectable disease.

**Conclusion:**

The previous report of such co-occurrence, together with our report and previous reports of nephrogenic rests, led us to wonder if there may be any causal relationship between these two rare entities. The wingless/integrated (Wnt) pathway, which is implicated in kidney development, is constitutively activated in approximately 15–20 % of all non-anaplastic Wilms tumors. Interestingly, the Wnt pathway was recently found to be perturbed in a zebrafish model of Cornelia de Lange syndrome. Mutations in cohesin complex genes and regulators have also been identified in several types of cancers. On the other hand, there is no clear evidence of an increased risk of cancer in Cornelia de Lange syndrome, and no other similar cases have been published since the fist one reported by Cohen, and this prompts to think Wilms tumor and Cornelia de Lange syndrome occurred together in our patient by chance.

## Background

Cornelia de Lange syndrome (CdLS, OMIM 608667) is the prototype for cohesinopathy disorders, which are developmental disorders with mutations in an evolutionarily conserved complex that functions in sister chromatin cohesion. However, the complex is also implicated in an increasing number of functions, including transcription regulation, DNA repair, chromosome condensation, and homolog pairing [1]. The CdLS phenotype is widely heterogeneous but characterized mainly by distinctive facial features, growth and cognitive retardation, limb defects, and a range of other malformations of the heart and kidney, among others [2]. Approximately 60 % of CdLS patients have mutations in *NIPBL* [3–6], and approximately 5 % have mutations in one of the other cohesin-associated genes, including *SMC1A*, *SMC3*, *HDAC8*, and *RAD21* [7–10]. Almost all of these mutations are *de novo*.

Genotype-phenotype correlations have been reported for these CdLS-associated mutations. For example, *NIPBL* mutations are typically found in patients with classical CdLS features, with missense mutations giving rise to milder phenotypes. In contrast, mutations in *SMC1A* or *SMC3* are associated with fewer structural anomalies and consistently with intellectual disability. Mutations in *HDAC8* and *RAD21* are now recognized as being associated with milder phenotypes without limb involvement and with atypical phenotypes with milder cognitive involvement and typical facial features [11–13]. Increased knowledge about the genetic basis of CdLS has led to an expansion of the phenotype and speculation that the prevalence of CdLS, first estimated to be 1.24/100,000 births, may actually be higher (~1/10,000).

CdLS is commonly associated with a wide range of renal abnormalities [2, 14], including nephrogenic rests [15, 16]. To the best of our knowledge, only one case of Wilms tumor (WT) and CdLS has been reported in the literature. The case was a post-mortem finding in a girl who died of bronchopneumonia at 7 months of age [17]. Here, we report a second case of the co-occurrence of WT and CdLS in a 3-year-old girl, the first in a living child. The tumor was detected by ultrasound examination at the age of 2 years that was performed as part of a routine exam because of the CdLS syndrome. This co-occurrence prompted us to question whether CdLS could have predisposed the patient to developing WT or whether the two entities co-occurred by chance.

## Case presentation

The patient, a 4-year-old girl, was the second child of a healthy, nonconsanguineous couple. The family history was negative for genetic diseases, and the child was born after a normal gestation by vaginal delivery. Her birth weight was 2.600 g (5^th^ percentile), her length was 47 cm (10^th^ percentile), and her occipitofrontal circumference (OFC) was 30.5 cm (<3^rd^ percentile). The following features were noted at birth: a high palate, synophrys, low-set hairline, a small up-turned nose, a single transverse palmar crease, and hypertrichosis of the face and the back. No major hand malformations were detected, and an examination of the kidneys and urinary tract showed no anomalies. The phenotype was mild (total score 14) according to the clinical score suggested by Selicorni et al. [6]. The standard karyotype was normal. A suspicion of CdLS was confirmed by molecular analysis of *NIPBL* (NM_133433), which revealed a c.4920 G > A *de novo* mutation. This variant has never been reported in the literature, ExaC, or the 1000 Genomes browser. The variant is considered disease-causing by prediction tools (i.e., mutation tester) with a high probability score. A perturbation of normal splicing is expected, in fact the mutation affects the last base of exon 24. Moreover, the variant was not present in the patient’s parents, confirming its pathogeneticity. A hyperechoic solid mass in the right kidney measuring approximately 3 cm at its maximum diameter was detected by renal ultrasound scan performed as part of a routine exam at the age of 2 years. The lesion lacked MRI contrast enhancement and initially thought to be benign. However, one year later, ultrasound showed that the mass had grown to a length of 5 cm. Computerized tomography (CT) characterized the lesion as a large enhanced mass protruding from the renal capsule that did not affect vessels or adipose tissue; these findings suggested that the lesion had a malignant nature. A Tru-Cut biopsy revealed non-anaplastic triphasic nephroblastoma, and the patient was treated pre-operatively according to the AIEOP-TW-2003 protocol (i.e., four courses of a regimen of vincristine and actinomycin D). Nephrectomy was then performed, followed by an additional 4 weeks of chemotherapy. At the last follow-up 19 months after treatment, the patient was healthy with no detectable disease, with hypertrophy of the contralateral kidney, 75^th^ percentile according to body surface area (BSA), and a normal glomerular filtration rate (128 mL/min/1.73 m^2^ BSA). At that time, the patient’s weight was 11.5 kg (50^th^ percentile for CdLS), height 92 cm (50^th^ percentile for CdLS), and OFC 42 cm (50^th^ percentile for CdLS) [18].

## Conclusion

Kidney malformations are commonly seen in CdLS. Selicorni et al. [14] reported a 41 % global incidence of renal abnormalities in pediatric CdLS patients. Although some evidence indicates premature aging in CdLS patients and mutations in cohesin complex genes and regulators have been identified in several types of cancers, the incidence of malignancy does not seem to be increased in CdLS patients compared to the general population [19, 20].

There are only single reports of different type of tumors co-occurring with CdLS, including suprasellar germinoma [21], papilloma of the chorioid [22], adenocarcinoma of the esophagus [23], hemangioendothelioma, and WT [17]. The last two were incidental findings at autopsy. The WT seemed to have been non-anaplastic on the basis of the microscopic histological description. The majority of these tumors (i.e., germinoma, hemangioendothelioma, WT) typically occur in childhood. The cohesin network is involved in gene expression during embryogenesis, and the CdLS phenotype clearly reflects the effects of cohesin network disruption on the embryogenesis of various organs and tissues.

Wilms Tumor, or nephroblastoma, is the most common renal tumor in childhood. In 1988–1997, the age-standardized incidence rate of childhood renal tumors in Europe was 8.8 per million, with WT accounting for 93 % of cases [24]. Several genetic syndromes are related to a specific, and sometimes quantified, increased risk of WT, but the extreme rarity of the co-occurrence of WT with particular syndromes makes it difficult to establish a direct relationship between the two [25, 26]. This may also be the case for CdLS and WT.

The disruption of at least three genetic pathways has been linked to tumorigenesis in WTs, partially explaining its heterogeneity [27]. The wingless/integrated (Wnt)/β-catenin pathway (canonical Wnt pathway) [28–31] is constitutively activated in approximately 15–20 % of all non-anaplastic WTs; abrogation of the pathway can promote tumorigenesis and nephrogenic rest development [32]. WNT and related signaling pathways also play a crucial role in kidney differentiation and the initiation of nephrogenesis [27].

Pistocchi et al. [33] examined the effects of perturbations in the canonical WNT pathway in a zebrafish model of CdLS with a focus on its expression in the developing central nervous system. These experiments suggested that the WNT pathway is downregulated by a loss-of-function of *NIPBL*. The interaction between cohesin and WNT pathways is of great interest because WNT plays a role in the non-anaplastic forms of WT, which is the histological type in the patient reported here. Unfortunately, we could not retrieve the biopsied tissue.

To the best of our knowledge, this is the second report of WT associated with CdLS and the first report in a living patient. We explored whether the co-occurrence is stochastic or represents one possible scenario of kidney involvement due to CdLS. The latter hypothesis may be supported by the known role of the cohesin network in embryogenesis and by some reports of nephrogenic rests in CdLS [16]. Notably, nephrogenic rests are considered potential direct precursors of WT [34]. These observations led also to the hypothesis that WT tumorigenesis results from postnatal retention and dysregulated differentiation of blastemal elements in the kidney. Finally, a potential association between CdLS and WT may be underestimated because of spontaneous WT resolution, a misdiagnosis of nephrogenic rests, or a milder misdiagnosed CdLS phenotype. However, no other similar reports have been published in recent decades, and nephrogenic rests are found in 1 % of unselected pediatric autopsies [35, 36]. This rather prompts to think WT and CdLS occurred together by chance.

In conclusion, we reported the unusual co-occurrence of CdLS and WT, raising questions about an increased risk of WT development in patients with CdLS. Larger population studies are needed. To the best of our knowledge, WT has not been reported in cohesinopathies other than CdLS. Ultrasound screening during childhood is still indicated in CdLS because of the high prevalence of urogenital anomalies.

## Abbreviations

CdLS, Cornelia de Lange Syndrome ; WT, Wilms tumor; CT, computerized tomography ; Wnt, wingless/integrated 
